# Sensory processing in medically unexplained pain syndrome. A systematic review

**DOI:** 10.3389/fpain.2025.1584227

**Published:** 2025-06-06

**Authors:** Nicole Quodling, Norman Hoffman, Frederick Robert Carrick, Monèm Jemni

**Affiliations:** ^1^ The Carrick Institute, FL, United States; ^2^ Coelevate Chiropractic, Adelaide, SA, Australia; ^3^ College of Medicine, University of Central Florida, Orlando, FL, United States; ^4^ Burnett School of Biomedical Science, University of Central Florida, Orlando, FL, United States; ^5^ MGH Institute for Health Professions, Boston, MA, United States; ^6^ Centre for Mental Health Research in Association with the University of Cambridge, Cambridge, United Kingdom; ^7^ Faculty of Physical Education, Ningbo University, Ningbo, China

**Keywords:** central sensitization, fibromyalgia, complex regional pain syndrome, neuropathic pain, vision, audition, olfaction, touch

## Abstract

Chronic pain is inherently multifactorial, with biological, psychological and social factors contributing to neuropathic pain (NP) and central sensitization (CS) syndromes. Comorbidity between functional disorders and the lack of clinical biomarkers adds to the challenge of diagnosis and treatment, leading to frustration for healthcare professionals and patients. The main objective of this review is to investigate the association between NP, CS syndromes and sensory processing disorders. A structured search was conducted on the PubMed database using the keywords Central Sensitization, Fibromyalgia, Complex Regional Pain Syndrome, and Neuropathic Pain, combined with the keywords Vision, Audition, Olfaction, Touch, Taste, and Proprioception. PubMed was chosen because it is accessible and user-friendly. Articles within the last five years, from 2018 to 2023, have been included. 380 studies on conditions of CS and sensory processing were identified. After applying inclusion and exclusion criteria, the number of retained papers was 78. There were a few emerging themes. Reduced sensory thresholds were found to be comorbid with chronic pain conditions, particularly those with a component of CS. Both cranial nerve and sensory evaluation examinations may prove helpful as potential biomarkers for diagnosis and for potential treatments.

## Introduction

Chronic pain represents one of the most critical public health problems, accounting for significant personal, social, and economic burdens (1). Chronic pain is inherently multifactorial, with biological, psychological and social factors contributing to NP and CS syndromes with considerable comorbidity between the different functional disorders (2, 3).

NP, as defined by the International Association for the Study of Pain (IASP), is “pain caused by a lesion or disease of the somatosensory nervous system” (4). This pain arises when a health condition impacts the nerves responsible for transmitting sensations to the brain, making it distinct from other types of pain. It can affect any nerve in the body, with some nerves more commonly impacted than others. Notably, diabetes-related neuropathy accounts for about 30% of all nerve pain cases. NP can vary in intensity, ranging from mild to severe, and may be persistent or fluctuate over time, affecting any part of the body (5).

The IASP further elaborates that NP requires “a demonstrable lesion or disease that satisfies established neurological diagnostic criteria” (6). It involves complex processes such as sensitization and alterations in brain connectivity (7, 8), initiated by various pathophysiologies including peripheral nerve injury, central nervous system injury, viral infections, tumours, and metabolic disorders (9, 10). This condition persists for at least three months or beyond the expected healing time (11–14).

NP syndromes are notably refractory to treatment and cause significant suffering (10, 15). The prevalence of NP is estimated to be between 6% and 20%, leading to high costs at both individual and societal levels (6, 10, 15, 16) and a decline in quality of life (17).

CS occurs when the patient's nervous system is persistently in a high-activity state, leading to an exaggerated response to pain stimuli. This condition, also known as centralized pain, central pain, or widespread/diffuse pain, is a syndrome influenced by both genetic and environmental factors (18). CS involves the amplification of neural signalling and dysfunction in neurophysiological mechanisms that increase neuronal responses to both noxious and non-noxious stimuli. It is a critical mechanism in chronic pain maintenance (7, 19–21). The underlying factors contributing to CS are complex, individualized, and poorly understood (21). CS is characterized by hypersensitivity to mechanical stimuli, a lowered pain threshold, prolonged pain after the stimulus has been removed, and significant increases in the excitability of nociceptive neurons (21, 22).

CS is inferred from symptoms like allodynia or hyperalgesia without a clear pattern of aggravating or relieving factors and is not in a dermatomal distribution (7, 14, 23). Centralized pain is associated with mood changes, fatigue, cognitive disturbances, sleep changes, catastrophizing, and often comorbid major depressive disorder or generalized anxiety disorder (24). Centralized pain affects between 5% and to 30% of the general population, with fibromyalgia (FM) or migraine being common conditions within this group (25).

Pain severity is often measured through subjective reports, while objective biomarkers that may guide diagnosis and treatment are lacking (15). This leads to ambiguity in diagnosis, difficulties in quantification, reliability and comparability, and uncertainty in understanding its physiopathology (26). There is significant interest in the field, as the biopsychosocial contribution, the lack of biomarkers and ineffective treatments frustrate clinicians and patients alike (14, 27). The clinical description is based on subjective report, history taking, clinical examination, and quantitative sensory testing (QST) (6, 28). Patients with CS syndromes report multiple sensory hypersensitivities, yet environmental sensitivity is not measured as part of the diagnostic process (27, 28). Pre-morbid or baseline sensory processing disorders seem to be a factor in developing CS pain in individuals with chronic musculoskeletal pain and may have been present from a young age (7). Assessing sensory function may prove helpful as diagnostic or predictive criteria and provide insight into potential treatment protocols. Only one paper utilized the SPQ to examine the relationship between FM and sensory processing disorder (27). No review was found to have extensively examined the potential link between chronic pain syndromes in general and sensory processing disorders.

The main objective of this review is to investigate the association between NP, CS syndromes and sensory processing disorders. This paper aims to explore simple definitions of how sensation works, progressing beyond the five traditional senses, to delve into newly recognized senses such as proprioception and equilibrioception.

## Method

Following the PRISMA framework, a structured search was conducted on the PubMed database using the keywords Central Sensitization, Fibromyalgia, Complex Regional Pain Syndrome, and Neuropathic Pain, combined with the keywords Vision, Audition, Olfaction, Touch, Taste, and Proprioception. PubMed was chosen because it is accessible and user-friendly. Articles within the last five years, from 2018 to 2023, have been included. 380 studies on conditions of CS and sensory processing were identified. After title and abstract screening, 138 studies were identified as meeting inclusion. These papers were then sorted into two categories—those primarily discussing sensory processing in pain syndromes and those discussing treatment options. The number of papers discussing sensory processing was 78. Papers were excluded if they were animal studies, investigated tissue damage, disease processes or addiction, were conference proceedings or non-English. Only a few relevant reviews that add specific details on the condition have been retained, to summarise evidence from different outcomes, conditions and populations. As this study is a systematic review of previously published research rather than a clinical trial or experimental investigation, the risk of bias was assessed independently by at least two reviewers. Discrepancies were resolved through discussion or adjudication by a third reviewer. A colour-coded system was used to visually flag studies based on their risk level (e.g., low, moderate, or high), supporting transparent identification of studies warranting further discussion.

## Results and discussion

**Figure 1:**
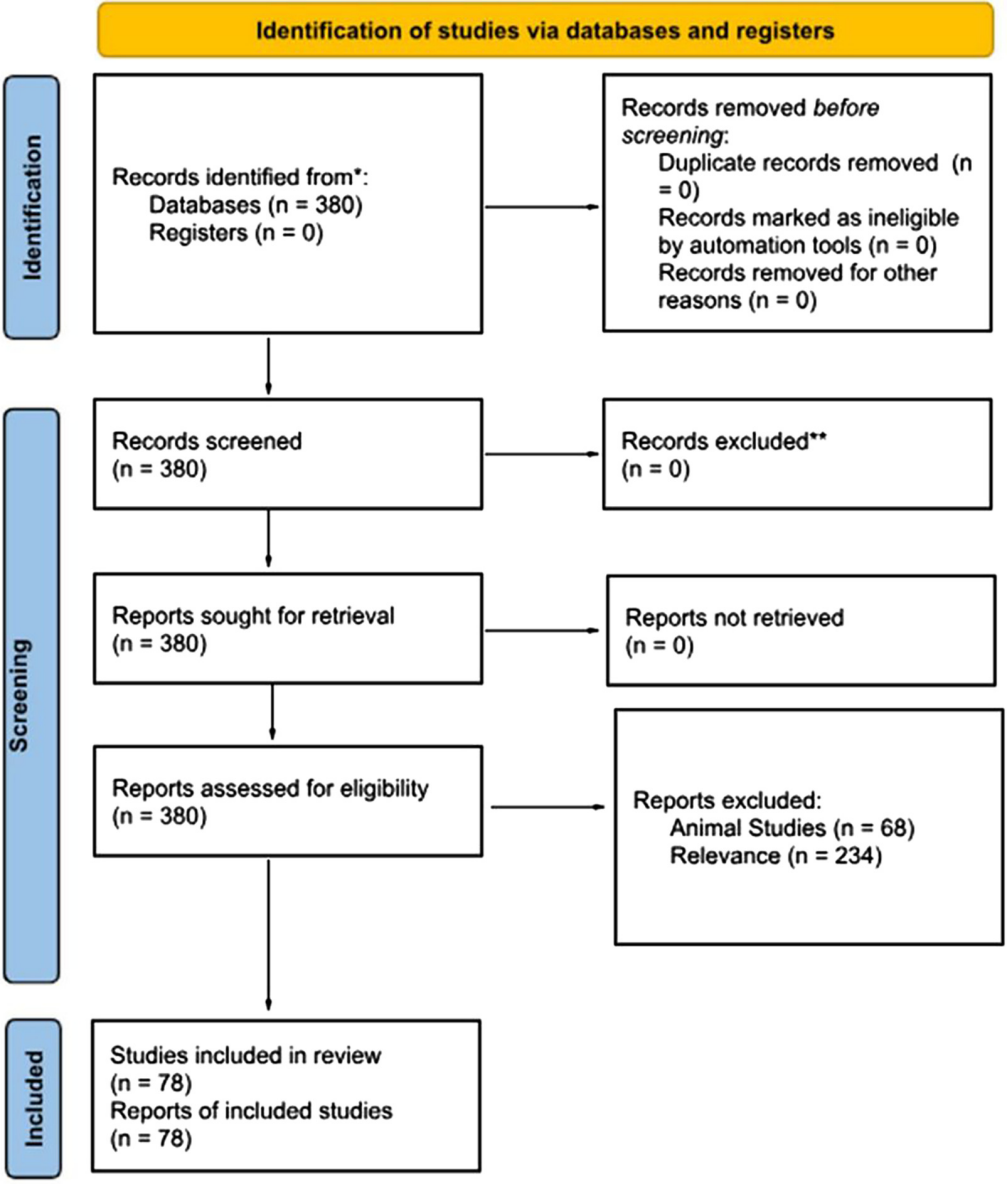
PRISMA 2020 flow diagram for new systematic reviews which included searches of databases and registers only. For more information, visit http://www.prisma-statement.org/.

380 studies on conditions of CS and sensory processing were identified. After title and abstract screening, 78 studies were identified as meeting inclusion in this paper (Figure 1).

Findings are summarized in Table 1.

**Table 1:** Sensory findings by condition.

Author	Condition	Sensation examined	Sensory findings
Alshahrani and Reddy (29)	Fibromyalgia	Proprioception	Cervical JPS and limits of stability were impaired, mediated by kinesiophobia
Aroke et al. (8)	Chronic pain	Taste	TRPV1 and TRPA1 associated with taste and mediating pain sensitivity
Augière et al. (30)	Fibromyalgia	Touch	Hyperalgesia. No difference in sensitivity to non-noxious tactile stimuli
Basha et al. (31)	Central sensitisation (Vulvodynia)	Touch	Lower forearm pain thresholds to cold and heat stimuli. Lower vestibular pain thresholds to cold (and pressure).
Basso et al. (32)	Neuropathic pain and central sensitisation (Tinnitus)	Audition	Reduced hearing ability, hearing-related difficulties.
Bellan et al. (33)	CRPS	Proprioception	Ability to localise limb unaffected in CRPS. Dissociation between implicit and explicit neural processes suggests neglect-like characteristics.
Berryman et al. (34)	Fibromyalgia	Audition	Sensory suppression (PPI) is intact, but sensory facilitation (PPF) is increased.
Berwick et al. (35)	Fibromyalgia	Touch	Decreased pressure threshold with pain for several days. Aftersensations with brushstroke stimulation.
Boehme et al. (11)	Fibromyalgia	Touch	Slow (CT-optimal) and fast (CT-suboptimal) brushing rated less pleasant
Brun et al. (36)	CRPS	Proprioception	Altered sense of limb position and movement. Greater pain with limb movement
Clark et al. (7)	Central sensitisation (low back pain LBP)	Audition, olfaction, vision, touch, taste, proprioception	Preexisting sensory processing differences predispose to CS in LBP
Colorado et al. (25)	Central sensitisation (dry eye)	Vision	Hormonal changes over the menstrual cycle influence sensory systems in women
Delussi et al. (37)	Central sensitisation (migraine)	Olfaction	Osmophobia in both migraine and chronic tension type headache cases with anxiety
De Meulemeester et al. (38)	Central sensitisation (tinnitus)	Touch	Local mechanical hyperalgesia with tinnitus, increased with comorbid chronic idiopathic neck pain. Distant mechanical hyperalgesia with tinnitus + chronic idiopathic neck pain
Di Lernia et al. (13)	Central sensitisation and neuropathic pain	Touch, proprioception	Low interoceptive accuracy and confidence across chronic pain conditions. Interoceptive tactile stimulation effective in pain management
Di Pietro et al. (39)	CRPS	Touch	Poor tactile acuity on the painful limb
Dorris et al. (27)	Fibromyalgia	Audition, olfaction, vision, touch, taste	Greater sensory hypersensitivity to touch, vision, and smell
Dydyk and Givler (18)	Central sensitisation	Touch	CS associated with allodynia and hyperalgesia
Gossrau et al. (40)	Neuropathic pain [post herpetic neuralgia (PHN)] and CRPS	Touch	Increased pain perceived in PHN and CRPS with gentle brushstrokes. CT fibre function reduced in chronic PHN
Grayston et al. (41)	Fibromyalgia	Touch	High prevalence of small fibre neuropathy
Guerrero-Moreno et al. (14)	Central sensitisation (dry eye)	Touch	Corneal hypoesthesia after mechanical and thermal, stimulation
Habig et al. (42)	CRPS	Touch	CT-stimulation normalizes thermal pain thresholds but does not decrease pain
Harte et al. (43)	Central sensitisation (overactive bladder syndrome)	Touch, audition	Increased pressure pain and auditory sensitivity modestly associated with greater self-reported bladder pain
Hartmann et al. (44)	None examined	Touch, taste	Normative reference data for orofacial sensitivity collected
Houghton et al. (45)	None examined	Olfaction	Cognitive behavioural intervention (CBI) showed significantly increased odour thresholds
Hulens et al. (46)	Fibromyalgia, Central sensitisation (Chronic Fatigue Syndrome CFS)	Vision, audition, touch	Several disease characteristics similar in primary empty sella and FM/CFS
Hyland et al. (2)	Fibromyalgia, Central Sensitisation (CFS, irritable bowel syndrome IBS)	Proprioception, Vision, Touch	Pain, dizziness/balance problems, blurred vision described across syndromes
Jensen et al. (47)	Central sensitisation (LBP)	Touch	Supplemental tender point examination improved evaluation of LBP
Junad et al. (48)	Neuropathic pain (orofacial)	Taste	Duloxetine may aid in the recovery of taste following lingual and chorda tympani nerve injury
Kersch et al. (24)	Central sensitisation (paediatric chronic somatic pain)	Touch	Correlations between cutaneous somatosensory testing responses, deep pressure responses and biopsychosocial measures
Kim et al. (17)	Neuropathic pain	Proprioception	Lower handgrip strength appeared associated with symptoms suggestive of NP
Kim and Kho. (49)	Neuropathic pain (burning mouth syndrome)	Taste	Association described between taste alteration and pain
King et al. (50)	Central sensitisation (migraine)	Proprioception	Vestibular migraine patients abnormally sensitive to roll tilt during vestibulo-ocular reflex testing
Klein and Schankin (3)	Fibromyalgia, Central sensitisation (visual snow syndrome, migraine)	Vision, Touch, Audition, Proprioception	Visual or acoustic noise, vertigo, and somatosensory discomfort in perpetual disorders, with migraine as a common risk factor
Koca et al. (51)	Fibromyalgia	Audition	Highly frequent audiological symptoms with high hearing frequencies independent of disease severity
Kuttikat et al. (52)	CRPS	Touch	Altered cognitive processing of tactile stimuli
Le et al. (53)	Fibromyalgia	Audition	Significantly greater sensorineural hearing loss in FM
Lunden et al. (54)	CRPS	Touch	Paroxysmal pain was more prevalent in patients with thermal allodynia
Mason et al. (55)	Central sensitisation (knee pain)	Touch	Mechanical hyperalgesia associated with increased knee and global pain
Martín-Martínez et al. (56)	Fibromyalgia	Proprioception	Performance on 30 s chair stand test significantly related to fear of falling but not to number of falls
Martínez et al. (57)	Fibromyalgia	Proprioception	Increased vigilance to internal bodily cues
Mikkonen et al. (58)	Central sensitisation (LBP)	Proprioception	Postural sway parameters did not differ between pain-free controls and subjects with chronic LBP
Mingels et al. (59)	Central sensitisation (headache)	Proprioception	Spinal postural variability related significantly to extra-cephalic pressure pain thresholds
Mingorance et al. (1)	Fibromyalgia, central sensitisation (LBP)	Proprioception	Patients with chronic pain showed worse somatosensory sensitivity and motor function
Özsoy-Ünübol et al. (60)	Fibromyalgia	Olfaction, taste	Altered olfactory and gustatory function correlated with anxiety and depression
Peinado-Rubia et al. (26)	Fibromyalgia	Proprioception	Impaired dynamic and static balance impairment
Rasouli et al. (61)	Fibromyalgia, Central sensitisation (CFS)	Proprioception	Larger postural sway and insufficient control, with no significant differences between FM and CFS.
Rasouli et al. (62)	Fibromyalgia, Central sensitisation (CFS)	Proprioception	Insufficient postural control across both conditions. Fatigue but not pain correlated with postural control variables.
Reddy et al. (63)	Fibromyalgia	Proprioception	Cervical joint positional sense and postural stability impaired in FM with a moderate to strong relationship between joint positional sense and postural stability
Sempere-Rubio et al. (64)	Fibromyalgia	Proprioception	Impaired postural control worse when sensory inputs are altered but not correlated with lower limb strength
Sempere-Rubio et al. (65)	Fibromyalgia	Proprioception	Significantly lower QoL predicted by impaired postural maintenance and pain threshold
Shiro et al. (66)	CRPS	Vision	Some CRPS patients differed in visual attentional behavior toward the face and body
Tuncer et al. (67)	Fibromyalgia	Audition	Impaired hearing thresholds, lower resonance frequency values, and abnormal cVEMP and oVEMP indicating both auditory and vestibular system involvement
van den Broeke et al. (19)	Central sensitisation	Touch	Activity-dependent CS increases pinprick-evoked autonomic arousal measured by enhanced pupil dilation response
Verfaille et al. (68)	CRPS	Vision, Proprioception	CRPS display difficulties performing tasks requesting visuo-motor coordination
Villafaina et al. (69)	Fibromyalgia	Proprioception	Higher dual-task costs in overall sway and anterior/posterior sway
Wang et al. (70)	CRPS	Proprioception	Lower weighting of bimanual hand representation alleviated additional cutaneous input.
Wang et al. (71)	Central sensitisation (experimentally evoked)	Touch	Heightened multisensory sensitivity may be risk factor for deep-tissue pain sensitivity
Wang et al. (72)	Central sensitisation	Vision, olfaction, audition, touch, proprioception	Heightened multisensory sensitivity risk factor for altered CNS processing of sensory inputs, including pain

### Neuropathic pain syndromes

Nociplastic pain is associated with dysfunction of the somatosensory nervous system and may result in complex alterations in cognitive and emotional neural functions. Disrupted interoceptive processing is associated with the perception and maintenance of pain (13, 48), paraesthesia, and dysesthesia (16, 49). Since no pain biomarkers exist, the physical examination can only provide supporting evidence for a neurological lesion or disorder that could cause pain (6, 15).

Synaptic plasticity, underlying learning and memory, is a particular component of CS. Syndromes include FM, whiplash, temporomandibular disorders, non-specific low back pain (7, 21), vulvodynia (31) and migraine (25). Centralized pain is associated with mood changes, fatigue, cognitive disturbances, sleep changes, catastrophizing, and often comorbid major depressive disorder or generalized anxiety disorder (24).

Individuals with pain syndromes may experience proprioceptive and balance impairments, gait alteration, sensorimotor deficits and distortions of body representation, which could be caused by alterations in sensory processing (3, 21, 26, 28–30, 34, 60, 61, 64, 73) and a compromised habituation response to repeated stimulus exposures (74).

Dysfunction of the central, autonomic and peripheral nervous systems, alteration of neurotransmitters, endocrine and immune systems, oxidative stress, external stressors and psychological aspects have been implicated (28, 41, 46, 51, 53, 65, 67, 75, 76).

#### The Complex regional pain syndrome (CRPS)

The complex regional pain syndrome (CRPS) is an example of a NP syndrome. It is characterized by spontaneous or regional pain arising in one or more limbs, usually the upper limb, disproportionate to an inciting event and associated with trophic changes and sensory, motor, and autonomic dysfunction (39, 40, 42, 52, 54, 66, 68, 70, 77). Cognitive difficulties have been reported to affect the ability to represent, perceive and use the affected limb (52, 57, 68). Motor disturbances of the affected limb sometimes spread to the unaffected limb(s) associated with maladaptive “inter-limb coupling” (77). CRPS usually develops from a peripheral event, but its maintenance relies on neuroplastic changes within the central nervous system (39, 40, 52, 54, 66, 77). These deficits implicate distortions in body representation and are corroborated by the evidence of cortical reorganization associated with inflammation, autoimmunity, and genetic, structural and functional changes, although much remains unclear (40, 54, 70). The estimated incidence of CRPS is 5.5–26.2 cases per 100,000 people per year, with females affected more than males. CRPS occurs in approximately 2%–7% of patients who experience limb fractures, injuries or surgery (42).

#### Fibromyalgia

FM is a complex multifactorial condition of unknown aetiology characterized by chronic widespread pain, hyperalgesia and allodynia (28, 34, 41, 46, 51, 56, 57, 67, 73), leading to significant disability and reduced health-related quality of life (27–29, 41, 53, 56, 63, 64). Patients present with multiple sites of pain or tender points, fatigue, cognitive impairment, sleep impairment, and emotional or mood fluctuations (1–3, 26, 27, 35, 41, 53, 56, 57, 60, 61, 63, 67, 69, 78). Individuals with FM may experience proprioceptive and balance impairments, gait alteration, sensorimotor deficits and distortions of body representation, which could both be caused by alterations in sensory processing (3, 21, 26, 28, 30, 34, 41, 60, 61, 64, 73) Alshahrani & Reddy and a compromised habituation response to repeated stimulus exposures (74). Dysfunction of the central, autonomic and peripheral nervous systems, alteration of neurotransmitters, endocrine and immune systems, oxidative stress, external stressors and psychological aspects have been implicated (1, 28, 41, 46, 51, 53, 65, 67, 75, 76, 78). FM has also been reported to develop as a result of cervical spine injury but, the relationship between spine injury and FM remains unclear (78). The total prevalence of FM in the general population ranges from 0.2% to 11% and is most frequent in women (1, 3, 26, 30, 34, 35, 41, 51, 53, 56, 57, 63, 67, 73, 78).

#### Diagnosis and assessment

An adequate diagnostic method is required to determine functional deficiencies and achieve adequate therapy objectively (44). Several tests were utilized in the literature and included painDETECT (17, 35), The Central Sensitization Inventory (CSI) (21, 72), Quantitative Sensory Testing (QST) (6, 28, 30, 35, 40, 44, 48, 54, 55, 71), The Sensory Perception Quotient (SPQ) (27), the Fibromyalgia Impact Questionnaire (FIQ) (26, 79), the Body Perception Questionnaire (BPQ) (57) pain drawings (80) and the Tampa Scale for Kinesiophobia (TSK) (77). Pre-morbid assessments of high sensory sensitivity using QST is a predictor of CS (71).

#### The role of biomarkers

The National Institutes of Health (NIH) defines biomarkers as: “characteristics that can be objectively measured and evaluated as an indication of normal or pathogenic processes or pharmacological responses to a therapeutic intervention” (81). Research into potential biomarkers for medically unexplained pain, including chronic NP (82–84), CRPS (52, 85), FM (34, 86), and CS (81), is ongoing. The pursuit of pain biomarkers has mostly followed two general directions: serum markers (84, 86) and brain neuroimaging (15, 81, 83, 85). However, none of the analysed metabolites have been shown to be sufficiently reliable to create valid and reproducible testing (81, 86). The development of imaging provides objectivity and connects structural changes and potentially therapeutic or diagnostic information by highlighting the involved area of dysfunction. However, structural and functional changes in the neuraxis do not always correlate with pain perception (83). Neuroimaging is also expensive and currently unavailable for routine clinical use (81). Exploiting possible sensory processing issues within the clinical environment may provide screening biomarkers for further investigation (27, 28) and provide viable targets for non-opioid interventions and the development of mechanistic approaches to pain management (84). The approval of composite serological, imaging and clinical biomarkers, emerges as the way forward to improve diagnosis, subtyping, predictive and prognostic evaluation and the development of therapeutic options (15, 81, 83, 85, 86).

Few potential clinical biomarkers were examined in the literature. Vestibular migraine patients were proposed to be abnormally sensitive to roll tilt during vestibular ocular testing, potentially providing a biomarker that allows individual patients to be subtyped as vestibular or non vestibular migraineurs and facilitating individualised treatment (50). Compared with healthy controls, altered but highly variable tactile discrimination performance was shown across CRPS patients. Late latency responses could provide convenient biomarkers of abnormal perceptual decision-making mechanisms in CRPS to aid clinical detection and treatment (52). FM patients were shown to have similar startle responses to healthy controls but reacted more strongly to subsequent sound, demonstrating increased prepulse facilitation. This suggests that the reaction to new or surprising stimulation is overactive, indicating high sympathetic nervous system activity. It was suggested that, as prepulse inhibition was intact, therapies that calm the nervous system, such as vagus nerve stimulation, mindfulness, or music therapy, could be helpful for FM (34).

Objective biomarkers and classification by sensory profiles have been suggested to further understanding of underlying mechanisms, prognosis and validation of therapeutic efficiency for chronic pain conditions (15, 44, 54). The rationale for treatment aims to modulate central nervous system plasticity (34).

### Neuropathic pain mechanisms

#### Central sensitization and sensory processing

Functional disorders are diagnosed by symptoms after other biomedical causes of these symptoms are ruled out (2). Pain hypersensitivity involves multiple mechanisms, including CS, conditioned pain modulation, reward and motivation, epigenetic mechanisms and neuroinflammation, including microglial activation (24). Triggers such as stress, trauma, or environmental changes are associated with the onset, maintenance and exacerbation of the syndrome (3). NP and CS syndromes are characterized by spontaneous pain and almost always by sensory loss and sensory gain (11, 71). Patients display hypersensitivities to internal and external stimuli (22, 27, 28, 30, 34, 35, 45) so that daily life sensations, including noise, light, touch and smell, become aversive (3, 22, 72). Reduced sensory thresholds often exceed the predominantly affected sensory modality, including touch, visual or acoustic noise, odors, and proprioception (3, 28, 43, 71) to a degree that is distressing and painful (45, 67, 71, 72). Furthermore, generalized sensory sensitivity is associated with the presence of chronic overlapping pain conditions (72).

#### Interoception, sensory processing and associated brain changes

Pain is inherently interoceptive. Such differences also extend to the processing of interoceptive signals (13). FM patients are less able to perceive inner bodily sensations accurately, with interoceptive accuracy correlated with pain intensity, anxiety and depression. Intact unimodal processing of sensory information is essential for integrating these signals with other information, which is at the core of body representation and motor control (30).

The thalamus is a central component of all sensory networks implemented in filtering sensory input. Thalamocortical dysrhythmia has been discussed as the neuronal correlate of several syndromes of pain syndromes (3), most notably FM (30, 41) and CRPS (39). FM patients have demonstrated decreases in grey matter in the prefrontal cortex (11) which has reciprocal connections with the anterior cingulate cortex, amygdala, insula and ventral striatum, to make it well positioned to influence pain perception, including interindividual variability in negative affective responses to multimodal stimuli (15). The insula cortex integrates the forebrain structure involved in sensory perception, learning, and memory (76), playing a crucial role in evaluating and prioritizing stimuli and perceptual decision-making (3) and the psychosocial components of pain (76). Hypervigilance may not be limited to external sensory input but also involves an enhanced awareness of internal bodily cues (57).

Additionally, CS patients show higher activation in motor cortices and rate stimulation as more painful with reduced connectivity between the somatosensory cortex and medial frontal and prefrontal cortices, parahippocampal gyri, thalamus, and pons (3). These cortical areas are influenced by cognitive processing, which includes attentional demands and movement complexity (77). Pain-related central disturbances affect postural control, synergistic muscle activation and recruitment to maintain joint stability and movement in conditions of CS, including FM (1) and CRPS (70). Interestingly, these areas are not dedicated to the reception of the senses (11).

#### Transient receptor potential (TRP) channels

Two primary types of nerves can detect and transmit painful signals: unmyelinated C-fibers and myelinated A*δ*- fibers, which depend on electrical signals generated by ion channels, such as Transient Receptor Potential (TRP) channels (8). TRP are non-selective ion channels mediating the fluxes of various cations across the cell membrane. They are widely expressed in the nervous system including in the substantia nigra, hippocampal pyramidal neurons, hypothalamus, locus coeruleus and cortex to function as cellular sensors (22). Various cellular environmental stimuli such as chemicals, temperature (54), stretch/pressure, osmolarity, and pH (8, 75), activate TRPs to play a significant role in the five primary senses as well as the sense of pain (8, 22). Repeated, chronic activation of TRPA1 (22), TRPM8 and TRPV1 receptors can lead to upregulation and sensitization (22, 54, 75), which may result in a more robust cellular response to an activating substance.

Sensitization involves receptor hyperexcitability and perceiving an input as noxious, even from a standard or subthreshold, generally innocuous stimulus (22). As the role of TRPs in chronic pain and pain modulation emerges, many TRP channels have been examined as potential therapeutic targets for pain management (8, 14, 22, 75).

### Comorbidities of neuropathic pain

#### Vision

CS and concomitant sympathetic nervous system disorders have been associated with retinal nerve fiber thinning, decreased optic disc perfusion, blurred vision, visual field defects and diplopia (46, 78), dry eye (20, 25), photoallodynia, increased visual attention to symptomatology and decreased visual attention to other areas (3, 29, 66). CS can induce an exaggerated pain response when faced with visual illusions that involve sensory incongruence (33). Pinprick stimulation of areas of peripheral hypersensitivity elicits an increased pupil dilation response, making pupil size a possible sensitive measure for detecting the presence of CS (19, 41).

#### Audition

Audiovestibular complaints are often attributed to central hypersensitivity, despite standard hearing evaluations (28, 30, 34, 51, 64, 67, 73). Central nervous system gain leads to a greater risk for debilitating perceptual consequences, including hyperacusis, auditory hallucinations (3) or tinnitus, with or without vestibular symptoms (3, 50, 73, 79). Tinnitus can be associated with various physical and mental conditions, including chronic pain, depression and anxiety disorders (3, 32, 53, 79, 87). Patients with CS and nociplastic pain conditions can also demonstrate more significant rates of hearing loss (3, 28, 30, 43, 51, 53, 67), which is the most crucial predictor of tinnitus presence (79, 87). There is also preliminary evidence demonstrating that chronic neck pain and tinnitus correlates with findings of CS (38).

Anxiety is not only a predisposing factor for tinnitus but also a consequence of it, which can, in turn, impede habituation (32, 79). A more significant number of patients with tinnitus had chronically elevated cortisol levels. Glucocorticoid receptors are present in the inner ear so that cortisol can exert a direct influence on hearing detection thresholds with aberrant links between limbic and auditory system structures (79). The limbic and auditory systems interact at the thalamic level and modulate the perception of auditory signals. The conscious perception and distress of tinnitus seem to be influenced by connectivity patterns in the anterior cingulate cortex and left precuneus, the posterior cingulate cortex, and the right medial prefrontal cortex (3). The hearing and balance systems function interchangeably and should be evaluated in systemic conditions (67).

#### Olfaction

Olfactory hypersensitivity, anxiety and pain share common neural pathways and area activation and a possible functional association (3, 11, 30, 37, 60). Osmophobia, defined as fear, aversion, or psychological hypersensitivity to odors, is related to a broader sensorial hypersensitivity and symptoms of CS, such as greater chronicity, elevated anxiety and allodynia (30, 37). Conversely, olfactory loss is an early sign in diagnosing neurodegenerative disorders (22). Patients with FM can be hypersensitive to olfactory stimulations or show reduced olfactory bulb volume associated with decreased olfactory and gustatory function scores (30, 60).

#### Taste

Most individuals with altered taste function have primarily smell disorder, so smell and taste must be assessed concurrently (60). Neuropathic taste changes have been attributed to peripheral processing, central processing and microglial responses (48). TRP channels are located in nerve terminals, dorsal root ganglion, and taste buds, which play an essential role in pain and taste perception. TRP channelopathies have been associated with NP, inflammation, and reduced taste perception (8). Duloxetine has been shown to facilitate the recovery of partial nerve damage, helping to retrieve sensation and taste and providing evidence of a CS mechanism (48).

#### Touch

A pathognomic feature of CS is hypersensitivity to somatosensory stimulations (30, 34), which results in allodynia and hyperalgesia (27, 35, 88, 89). Other tactile misperceptions include decreased two-point discrimination and temperature detection (39, 52). Tactile allodynia, especially when accompanied by after-sensations, suggests likely CS (10, 16, 24) and is often reported in FM (27), NP and CRPS (54, 88). Blunt pressure pain thresholds are typically reduced as static mechanical allodynia, but in some cases, gentle brushstroke alone can induce dynamic mechanical allodynia (31, 35). At the cortical level, the anticipation of pain influences brain activity and increases sensitivity to somatosensory stimuli and a reduced ability to habituate to somatosensory stimuli (74).

Pain-sensitized patients characteristically show nociceptive system augmented responsiveness as a common feature (42, 73). Localized or diffuse hyperalgesia signifies regional or diffuse CS (24, 47). Hyperalgesia is often accompanied by allodynia (41). It is not restricted to tender points (30), although tender point examination may be used as a supplementary clinical test (47). Secondary hyperalgesia that is not associated with tender points is thought to result from CS (23). Pain after blunt pressure, tender point examination, pressure pain threshold, brushstroke and thermal assessment typically lingers for several days in FM and is correlated with clinical pain intensity (24, 35). Tender point examination in symptomatic patients can regularly cause long-lasting discomfort and is no longer required for diagnosis (35).

Persons with FM are hypersensitive to thermal stimulation, and show decreased rates of habituation (30, 74). Patients with chronic pain conditions can report more painful and longer-lasting painful aftersensations following exposure to noxious thermal stimuli (35), possibly indicating a pathology of small-diameter nerve fibers (42). There is a significant correlation between thermal allodynia and allodynia to light touch, as underlying hyperexcitability is likely to explain both phenomena (10, 54).

Poor tactile acuity has been observed in people with CRPS (33, 52) and reported to coincide with the distribution of pain (39). Poor tactile acuity could be related to aberrant perceptual representation of the environment or altered somatotopic mapping (52). Social touch is essential for interpersonal interaction and physical and social well-being (11) but, many patients with chronic pain conditions find generally pleasant stimuli intolerable, often inducing wind-up and increasing allodynia and hyperalgesia (11, 70). Uncomfortable aftersensations after brushstroke are associated with reports of reduced pleasantness in FM (11, 35). Mapping cortical activity during brush stroking reveals an inverted pattern of insula activity, inferring that anhedonia might be related to aberrant central nervous system evaluative processing. However, the finding of anhedonia does not exclude the possibility of abnormal signal processing of input from sensory afferents associated with small-fiber pathology (35). Small-fiber neuropathy is a disorder that selectively affects thinly myelinated A*δ* and unmyelinated C fibers that mediate pain, heat, and cold sensations (10, 41, 46) and affective aspects and rewarding aspects of touch (35, 40, 42). Social touch is essential for physical and social well-being, and its loss, tactile anhedonia, is an unmistakable feature of FM (11, 35). C Tactile (CT) stimulation can reduce experimental pain in healthy individuals; however, in patients with a reduced intraepidermal nerve fiber density, gentle stroking loses its pain-modulating properties as the pain modulating capacities of CT fibers might be too weak, or the feeling of chronic pain simply overrules normal CT fiber function (13, 42). Many chronic pain conditions show altered C-fiber innervation density, sensory loss, and pain sensitization (40), including FM (11, 41) and CRPS (54). Negative expectations and experiences towards the touch, such as in allodynia, could negatively affect touch perception (40).

#### Proprioception and vestibular symptoms

Vestibular symptoms of vertigo and unsteadiness are prevalent in CS (21), including migraine (50) and FM (26, 46) and probably result from an overreliance on visual and postural stimuli and reduced input from the central vestibular system (3). Impairment of proprioceptive processing has been observed in people with a range of persistent pain states (88). Proprioception is required for precise and synchronized action planning, joint stability, preserving static and dynamic balance, optimal posture maintenance (63, 64), and to perform tasks requiring visuomotor coordination (68). Body representations are blurred in pain, with alterations shown in motor and sensory cortical areas in CRPS (36, 68) and FM (29, 34, 73). Sensorimotor integration is crucial for planning our movements and their online monitoring and correction, but also to build a unified representation of our body (76, 90). Moreover, alterations in position sense have been associated with the severity of motor deficits (36).

Cervical joint position sense significantly contributes to functional balance mediated by the integrated function of proprioceptors present in the muscles, capsules, joints, and vestibular and visual stimuli. Changes in the proprioceptive signals are associated with cervicogenic dizziness in cervical disc degeneration (90) and FM (29, 63, 73). Cervical injury, pain and muscle fatigue can alter cervical proprioception from muscle spindles (63, 78), resulting in a sensory mismatch of vestibular and visual information, leading to dizziness and instability (89). Cervicogenic dizziness may also be caused by circulatory failure of the vertebral artery or cervical sympathetic nervous system disorders (78). Hand grip force (17), postural sway (61, 69) and cervical joint position sense (63) are reliable methods to measure physical capacity and muscle strength, the lack thereof being a good predictor for functional disability (73).

#### Posture and balance

Attention and sensory integration of visual and vestibular afferents are essential to produce appropriate motor output, including balance control (26, 57, 69). Alterations to vestibular, visual and somatosensory input could modulate the appropriate neuromuscular response where there is cervical disc degeneration (89) or in patients with FM (64). Alternatively, pain may force a patient to adopt a protective posture to protect the painful or threatened part of the body by constraining movement in the case of cervicogenic headache (59) or neuropathic pain (17), or the impact of pain on *γ* motor neuron activity can create long-term neurological adaptations of postural and motor behavior (1, 63).

Additionally, impaired postural stability has been linked to reduced muscle strength and stamina, decreased cognitive function, somatosensory integration, and pain processing (63, 64). Postural control impairment can affect balance, thus negatively affecting their quality of life (26, 29, 65). Poor balance has been considered a predictor of widespread musculoskeletal pain (1, 26, 29, 64, 69). The perceived lack of balance is related to the fear of falling, while objective balance is associated with the number of falls (56).

#### Tone

There is reduced muscle strength in FM and NP symptoms, primarily in female patients (17), both in grip strength, linked to a state of sarcopenia, weakness or dystonia, and in upper limb strength, linked to functional limb capacity (57, 65). Patients with muscle weakness have decreased muscle mass, incomplete muscle activation, decreased muscle spindle sensitivity, fewer sensory units, and fewer mechanoreceptors, all of which can affect the limits of stability (29). Hypermobility is significantly more frequent in FM than in control groups (73). Low handgrip strength is a clinically relevant predictor of poor patient outcomes, such as more extended hospitalization, impaired functional status, mental health problems, poor quality of life, and mortality (17).

#### Kinesiophobia

The fear of pain following movement or physical activity in patients with musculoskeletal pain can predispose to the development and aggravation of loss of muscle strength and mass (17). Soreness results in avoidance behaviors similar to those with other types of chronic pain (75). Pain is a highly salient signal of bodily harm and, thus, a strong motivator for learning (88). Poor physical fitness or fear of falling may also create avoidance of motor activities of daily living (56).

Kinesiophobia refers to the fear of movement brought on by activity or exercise and the catastrophic belief that such activity would result in damage or re-injury (29) and is frequent in patients with chronic musculoskeletal pain (17). Delayed onset muscle soreness (DOMS) usually leads to hyperalgesia, allodynia and ongoing muscle pain. Kinesiophobia may serve as a protective mechanism against discomfort and the worsening of pain by encouraging the avoidance of movement and the restriction of movement (29, 68). Protective behaviors are helpful in the case of acute pain because they avoid engagement with the pain. However, in chronic pain, this avoidance of interaction increases impairment (29). Fear of movement could impose additional cognitive demands, placing an extra burden on executing a movement task (77). This type of pain-related fear is less stimulus-bound and manifests itself instead as sustained anticipatory anxiety, which is prototypical for widespread pain disorders (88).

Kinesiophobia can cause mobility restriction in individuals with chronic pain and can change motor activation patterns, resulting in muscular weakening and atrophy (17, 59). These modifications can significantly alter the afferent proprioceptive input, affecting joint position sensation and stability limits (29, 36, 61) leading to further deterioration of postural control and fear of falling with a negative impact on endurance, muscle strength, flexibility, coordination, and quality of life (1, 29, 62, 69, 73).

#### Body image and neglect

Body image is a conscious representation of the body's appearance and our attitudes and feelings towards it, but is not used for action. Body schema is an unconscious representation with sensorimotor integration and is used for motor planning and execution, but may inform body image (36). Maintenance of the body schema depends on multisensory bodily inputs and may be altered in patients suffering from chronic pain (1, 21, 30, 33, 51, 52, 59, 62, 70). The severity of body perception disturbance has been linked to the reorganization of the primary and secondary cortical maps (66). Body image and schema are both tied to activity in the somatosensory and parietal areas, however, only body image is processed in the insula (36). Cortical changes in chronic pain conditions may cause alterations in spatial attention, leading to neglect-like symptoms (66). CRPS patients often describe their affected limb as feeling disconnected from their bodies, with difficulty initiating movements and performing actions consistent with the symptomatology of hemispatial neglect (68). Diminished interoceptive accuracy has been associated with depression and alexithymia (57), CRPS, FM, and NP disorders (13). Sensorimotor integration is crucial for planning movements and building a unified representation of our body (73). Individuals with acute or chronic pain report higher sensory disturbances in the presence of sensorimotor conflicts compared to pain-free individuals (12, 33, 61, 62). This may relate to the inability of incoming sensory inputs to adequately update somatic long-term memory (57).

## Clinical applications

Premorbid contexts may be related to the onset of CS. CS pain often develops in the context of sensory processing differences related to learning difficulties, sensitivities and trauma, and personal characteristics of low confidence and control (7). Sensory overload can significantly impact the quality of life (27). Although various aetiologies can cause CS, the symptoms and characteristics of pain are influenced by pathophysiological mechanisms rather than aetiology, with important therapeutic implications for personalized treatment (1). With the recognition that healthcare professionals consistently underestimate pain compared to patients, there is an increasing movement toward person-centered assessment of chronic pain conditions (27). The persistence of symptoms and refractoriness to treatment could be due to the central changes not sufficiently influenced by conventional approaches. Interventions integrating somatic, physical, and emotional factors should be considered when developing clinical programs (1), although the effect of most therapies is modest (34). There is an unmet need to characterize chronic pain patients regarding underlying mechanisms to aid early detection and treatment (52).

## Limitations

Despite the structured search strategy and specific eligibility criteria and goals, this review has limitations, related to methodological inconsistencies, study heterogeneity, low subject numbers and the reliance on self-reported data. Although this review was based on an extensive literature review, the study was limited by the selectivity of searches and databases. The findings of all conditions were assessed concurrently, with an assumption of a central origin, however a large proportion of studies examined CS, particularly FM, and proprioceptive deficits, and results may not be generalisable. The lack of biomarkers in medically unexplained pains leads to lack of consistency in measurement, assessment and reporting, further complicating comparison. Further studies are required with large study numbers, subgrouping of pain syndromes and homogenized methodology.

## Conclusion

The objective of this review was to investigate the association between NP, CS syndromes and sensory processing disorders. Objective biomarkers for medically unexplained pain syndromes are sadly lacking, thwarting reliable diagnosis and treatment strategies. Multiple sensory hypersensitivities are often reported and in conditions of CS, and could contribute to the diagnostic process and assessment of treatment success. Pre-morbid sensory processing disorders may be a risk factor for the development of chronic pain syndromes. Assessing sensory function through cranial nerve and other neurological examinations may prove helpful as diagnostic or predictive criteria and provide insight into potential treatment protocols.

## Recommendations and future directions

Considering the above limitations, the scientific community can move closer to developing more precise diagnostic markers and tailored treatment strategies for individuals suffering from NP and CS syndromes by pursuing the following:

### Development of standardized protocols

To enhance comparability and reproducibility, future research should adopt standardized methodologies for assessing NP, CS, and sensory processing (e.g., consistent inclusion/exclusion criteria, uniform pain assessment scales, and validated sensory evaluation tools).

### Integration of objective biomarkers

Given the limitations of subjective reporting, studies should explore and validate objective measures—such as cranial nerve evaluations, quantitative sensory testing (QST), and neuroimaging techniques—to identify and confirm the presence of CS and NP mechanisms.

### Longitudinal and interventional studies

Further longitudinal research is needed to clarify the natural progression of CS syndromes and to establish causal relationships. Interventional trials that incorporate both pharmacological and non-pharmacological approaches (e.g., sensory re-education, cognitive-behavioral therapy, neuromodulation) could provide deeper insights into effective management strategies.

### Personalized medicine approaches

Recognizing the heterogeneous nature of NP and CS syndromes, future efforts should focus on personalized treatment plans that account for individual differences in sensory processing, comorbidities, and psychosocial factors.

### Collaborative, multidisciplinary research

Scientists, clinicians, and specialists from fields such as neuroscience, psychology, physiotherapy, and pain medicine should collaborate to develop comprehensive models of chronic pain. This interdisciplinary approach can improve diagnostic accuracy and therapeutic outcomes.

### Focused investigation of high-risk groups

Targeted studies of populations underrepresented in the current literature—such as pediatric, geriatric, or culturally diverse groups—would help clarify how central sensitization and sensory processing manifest across different demographics.
